# Rapid Onsite Evaluation: A Prospective Observational Study of Endobronchial Ultrasound-Guided Transbronchial Needle Aspirates to Expedite Diagnosis

**DOI:** 10.3389/bjbs.2024.12938

**Published:** 2024-11-14

**Authors:** Carolyn Mercer, Kate Brackenborough, Colette Davidson, Tosia Nisar, Behdad Shambayati, Rupinder Cunningham, Ben Howarth, Anita Jayadev

**Affiliations:** ^1^ Respiratory Medicine, Wexham Park Hospital, Frimley Health NHS Foundation Trust, Slough, United Kingdom; ^2^ Respiratory Medicine, Frimley Park Hospital, Frimley Health NHS Foundation Trust, Frimley, United Kingdom; ^3^ Cytopathology Department, Berkshire and Surrey Pathology Services, St Peter’s Hospital, Chertsey, United Kingdom

**Keywords:** rapid-onsite evaluation (ROSE), EBUS, adequacy, lung cancer pathway, cytopathologist

## Abstract

Biomedical scientists (BMS) can report sample adequacy during EBUS TBNA using rapid on-site evaluation (ROSE). Despite being able to report exfoliative samples such as pleural fluid cytology and bronchial washings, they are usually not permitted to offer a preliminary diagnostic impression of EBUS TBNA samples. Experienced biomedical scientists can provide a reliable diagnostic impression during ROSE for EBUS samples, with sensitivity and specificity comparable to cytopathologist reporting. This work represents an advance in biomedical science because it provides evidence a BMS can safely and accurately provide a real time cytopathological impression from EBUS TBNA sampling, which could positively impact patient pathways.

## Introduction

Lung cancer is the leading cause of cancer deaths in the United Kingdom (UK), accounting for one in five cancer deaths [1]. Endobronchial ultrasound guided transbronchial needle aspiration (EBUS-TBNA) is the first-line approach for investigation of radiologically suspicious lymph nodes or lesions adjacent to the central airway for the diagnosis and staging of lung cancer [2]. The use of rapid on-site evaluation (ROSE) of transbronchial needle aspirates during bronchoscopy remains controversial [3]. It involves production of a slide in the bronchoscopy suite for rapid staining then evaluation by a trained biomedical scientist (BMS) or cytopathologist to provide an interpretation of sample adequacy. Choi et al have defined adequacy as potentially using up to four sequential criteria: tissue core size, the presence of malignant cells, Microscopic Anthracotic Pigment (MAP), and Lymphocyte Density (LD) ≥40 cells/field (area viewed at ×40 magnification) [4]. Using these parameters together significantly increased the sensitivity and accuracy rates of ROSE [4]. Evidence suggests ROSE decreases the number of needle passes required [5, 6], minimises the need for additional procedures [6–8], reduces the complication rate [6] and subsequently reduces cost [8, 9]. The evidence regarding sample adequacy and diagnostic sensitivity is mixed [10–12]. In the era of targeted therapy for lung cancer the use of ROSE can ensure the sample is sufficient for ancillary studies including immunohistochemistry and molecular testing [5]. However some operators and pathologists may feel that ROSE compromises the amount of tissue available for these additional tests [13].

At present, where available in the UK, ROSE is utilised to determine sample adequacy and is not typically employed as a diagnostic tool. This in large is due to the fact BMSs are not currently permitted to report a diagnostic impression on EBUS specimens during ROSE, only to report on adequacy of the sample. Combined with studies showing that there is no statistically significant increase in diagnostic yield with ROSE [14], means it is not uniformly used in EBUS centres. As with use of ROSE for thyroid FNA, many operators therefore feel the additional resources required and the associated cost are not justified [15]. However, considering challenging healthcare pressures in order to meet current recommended targets, it may be that the use of ROSE in EBUS has not been maximised to its full potential and it is time to review the current model. The UK National Optimal Lung Cancer Pathway recommends that patients with lung cancer symptoms should receive a diagnosis or exclusion of cancer within 28 days of referral [16]. The lung cancer clinical expert group from NHSE (National Health Service England) released EBUS quality and performance standards in 2019, recommending pathological results are received within 5 days of EBUS sampling [17]. Use of ROSE during EBUS-TBNA could help to reach and maintain those targets.

An experienced cytopathologist performing ROSE poses a significant economic and workforce burden for many institutions, which can limit its application, especially given 78% of pathology departments in the UK have reported consultant vacancies [18]. There appears to be a shift in approach, with the Royal College of Pathologists acknowledging that appropriately qualified staff can offer a preliminary diagnosis [19]. A joint position statement from the Royal College of Pathologists and the Institute of Biomedical Sciences (2023) states that “in some services, a provisional diagnosis may be offered at ROSE, if this is deemed necessary for immediate patient management – for example, a diagnosis of small cell carcinoma leading to same-day initiation of chemotherapy” [20]. Of note a trained and suitably qualified BMS is able to report exfoliative samples (cells that have been shed, scraped or brushed from a tissue surface) including pleural fluid cytology and bronchial washings in the UK. With evidence that a trained BMS undertaking ROSE of EBUS-TBNA minimises the puncture number and overall cost (by reducing need for repeat or further procedures) without compromising diagnostic accuracy; there is an argument that, with appropriate training, they should be permitted to report a provisional cytopathological diagnosis [6, 8, 17]. The aim of our study is to evaluate the reliability with which an experienced BMS can provide a provisional diagnostic impression of EBUS-TBNA samples compared to the ‘gold standard’ of cytopathologist diagnosis, and therefore whether undertaking ROSE can help expedite lung cancer diagnosis.

## Methods

### Patients and Procedures

A consecutive series of adult patients (greater than 18 years in age) from two independent hospitals in England undergoing EBUS-TBNA were included in the study. The procedures were carried out by multiple respiratory consultants and performed according to the British Thoracic Society Guidelines for advanced diagnostic and therapeutic flexible bronchoscopy in adults [21]. The decision regarding which lesions (lymph node, lung nodules/masses, or both) to sample were made according to best practice guidance and following recommendations from the Lung Cancer Multi-Disciplinary Team (MDT) at these two hospitals in Frimley Health NHS Foundation Trust. After the procedure, the total number of lymph nodes biopsied, number of passes and complications were recorded for all patients.

Due to clinical pressures, staffing issues and restrictions secondary to the COVID-19 pandemic, it was not possible for a BMS to be present to undertake ROSE at every procedure. Therefore, patients randomly either had or did not have ROSE performed at the time of their EBUS. For those procedures in which ROSE was performed the BMS reported adequacy in real time and advised the operator when they felt there was adequate material for a cytological diagnosis. As current guidelines do not permit them to provide a cytological impression, only adequacy is communicated to the respiratory consultant performing the procedure. In the first half of the study, we analysed the accuracy of adequacy reporting by the BMS. Once satisfied with the standards of adequacy reporting, the second half of the study commenced whereby the BMS independently recorded their impression of the underlying diagnosis, separately to the adequacy report. Their opinions were divided into four groups: Malignant, Granulomatous Inflammation, Reactive or Other (e.g., suspected lymphoma). This information was then submitted to the EBUS operators at the end of the procedure, who collected all data which was held securely. The cytopathologist was blinded to the BMS’ diagnostic opinion and the BMS was blinded to the cytopathologists impression and report. Both BMS and cytopathologists used the same slides prepared by rapid stains to report. As the cytopathologist is not present at the EBUS procedure, the BMS impression was easily blinded to them and similarly the BMS is not present at the time of the cytopathologist reporting so all data was collected and analysed by the respiratory team.

### Sample Handling and Pathologic Diagnosis

The non-ROSE group had no cytopathological examination at the time of their procedure. TBNA samples were collected, placed in formalin and later prepared in the laboratory for review by a cytopathologist. The ROSE group had ROSE performed by an **experienced** BMS. The locally agreed standards for “**experienced**” BMS were defined as: minimum qualification.

Advanced Specialist Diploma (ASD) and Diploma of Expert Practice (DEP) in NonGynaecological cytology of the UK Institute of Biomedical Sciences (IBMS); at least 2 years experience of ROSE for EBUS-TBNA and 100 supervised cases by a consultant BMS. The aspirates were expressed onto a glass slide, smeared and allowed to air dry. Rapid staining was performed using Diff-Quik (Rapid Romanowsky staining kits, widely available from commercial companies). The stains used were May-Grunwald-Giemsa, made up of acidic and basic dyes of eosin Y and methylene blue. The BMS evaluated the cell material on the glass slide to determine adequacy of the sample and record a cytological impression. Adequacy was defined by the presence of malignant cells or granulomas and Lymphocyte Density, LD ≥40 cells/field, in keeping with standards of practice in other institutions utilising ROSE for EBUS [22]. Where ROSE was available, the BMS submitted their adequacy report (NOT diagnostic impression) to the cytopathologist, as per standard procedure.

### End Points and Statistical Analysis

The primary outcome measure for the study was adequacy and cytological impression, i.e., diagnostic sensitivity, of an experienced BMS compared to board certified consultant cytopathologist with ROSE. Secondary outcomes included time to formal cytopathological diagnosis, diagnostic yield, number of stations sampled and need for repeat procedures.

Microsoft Excel and IBM SPSS Statistics Version 28.0.0.0 (IBM, United States) were used for statistical analysis. Continuous variables were summarised as mean and standard deviation (SD), and analysed using an independent *t*-test. Categorical variables were analysed using Pearson’s χ^2^ test or Fischer’s exact test. Sensitivity and specificity calculated with a 95% confidence interval (CI). All tests were two-tailed, and a *P*-value of 0.05 or less was considered statistically significant.

## Results

A total of 397 patients across the two hospital sites underwent EBUS-TBNA for evaluation of hilar/mediastinal lymphadenopathy from May 2018 to April 2021. The mean age of patients was 62.49 years (SD 15.41). Males comprised of 59.91% of the population. The final formal cytopathologist diagnosis is summarised in Table 1. These comprised of adenocarcinoma (6.1%), benign lymphoid tissue (26.2%), granulomatous inflammation (24.7%), insufficient (8.8%), lymphoma (0.5%), non-small cell lung cancer (11.8%), other (3.0%) (inflammatory changes, necrosis) other malignancy (5.5%), small cell lung cancer (7.3%), squamous cell cancer (6.1%).

**TABLE 1:** Final Diagnosis of EBUS-TBNA in ROSE vs. Non-ROSE Cases.

Final diagnosis	ROSE	Non ROSE	Total
	n = 298	n = 99	n = 397
Malignant			
Adenocarcinoma	23	1	24 (6.1%)
Non small cell carcinoma	38	9	47 (11.8%)
Small cell carcinoma	23	6	29 (7.3%)
Squamous cell carcinoma	19	5	24 (6.1%)
Lymphoma	2	0	2 (0.5%)
Other Malignancy	19	3	22 (5.5%)
Benign			
Benign lymphoid tissue	80	24	104 (26.2%)
Granulomatous inflammation	66	32	98 (24.7%)
Other	6	6	12 (3.0%)
Non diagnostic			
Insufficient sample	22	13	35 (8.8%)
Total	298	99	397 (100%)

**FIGURE 1:**
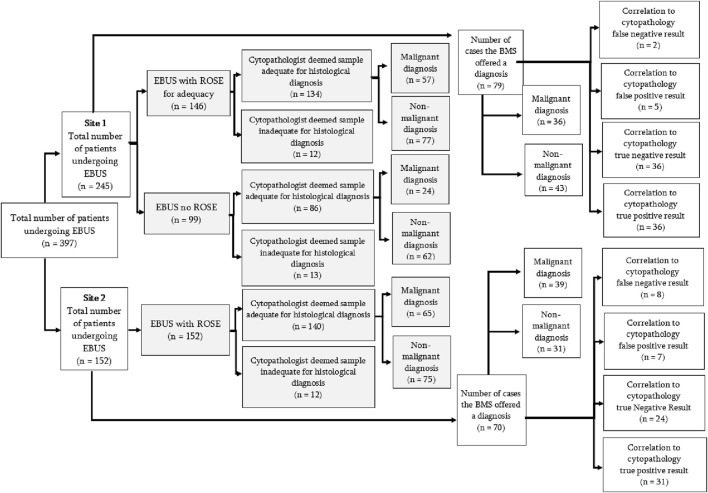
Flow diagram of all EBUS samples analysed. Adequacy analysis for the first part of the study is shown by the shaded boxes. The second part of the study, analysing accuracy of diagnostic impression by BMS, is represented by the non-shaded areas.

Two hundred and ninety eight patients had EBUS-TBNA evaluated by ROSE to confirm if the sample obtained was adequate. The BMSs correctly identified if a sample was adequate (number of cases in which sample was diagnostic/total number of cases) for histopathological analysis in 270 cases (90.6%). See Figure 1. There was no statistical significance in sample adequacy between ROSE and non-ROSE cases (*p* = 0.290). Diagnostic yield for all diagnoses (proportion of diagnoses achieved/patients undergoing EBUS-TBNA) in patients who had ROSE at EBUS-TNBA (n = 298) was 92.62% in comparison to 86.86% in patients who did not have ROSE (n = 99). This difference however was not statistically significant (*p* = 0.80).

**FIGURE 2:**
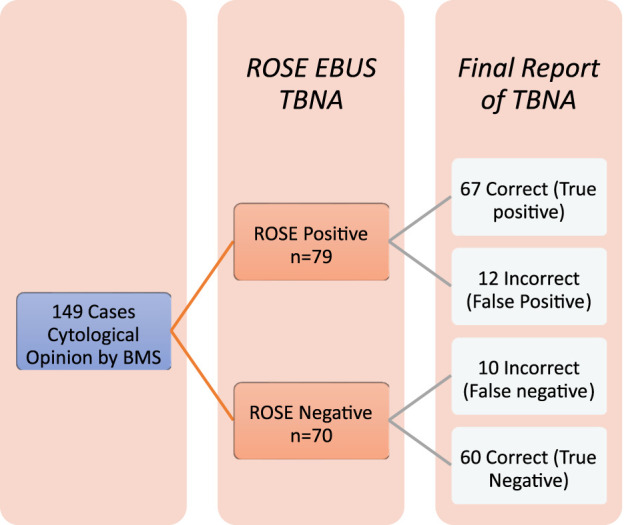
Accuracy of diagnoses of ROSE EBUS-TBNA by BMS.

A subsequent prospective analysis of a further 149 patients who had a cytological impression recorded by the BMS found that compared to the gold-standard reporting by the cytopathologist which is current accepted practice, Site 1 had a site-specific sensitivity of 94.7% and specificity of 87.8% (note site 1 performed more EBUS procedures). Site 2 had a sensitivity of 79.5% and specificity of 77.4% when comparing BMS cytological impression to that of the final cytopathologists report. The BMS diagnosis was accurate (true positives + true negatives/all cases) [8] in 85.23% of cases, yielding an overall sensitivity of 87.01%, specificity of 83.33% with a negative predictive value and positive predictive value of 85.71% and 84.81%, respectively (See Figure 2).

The mean number of stations sampled during EBUS in ROSE cases was 1.48 (SD 0.78) in comparison to 2.17 (SD 0.97) to non-ROSE cases. This was statistically significant with a *p*-value < 0.001. There was no statically significant difference with regards to the need for repeat procedures between the ROSE (n = 27/298) and non-ROSE (n = 4/99) groups (*p* = 0.107) and also no difference if the samples obtained during EBUS-TBNA were sufficient for immunohistochemistry (*p* = 0.129).

The mean time to the formal cytopathology report for all diagnoses was 7.86 days (SD 4.05, n = 386). There was no statistically significant difference in time taken for formal cytopathology report in the ROSE (mean = 7.87, SD = 4.85, n = 297) vs. non-ROSE (mean = 7.84, SD = 3.12, n = 89) cases (*p* = 0.962). The average time from date of referral to a final diagnosis was 15.94 days (SD 9.54, n = 193). When the cases were separated into malignant and non-malignant diagnoses, the time to the formal pathology report was 9.59 days and 6.79 days, respectively. This difference was statistically significant with a *p*-value of <0.001.

## Disscusion

EBUS-TBNA as a minimally invasive procedure has become the first-line approach to evaluate intrathoracic masses and mediastinal lymph nodes present in both non-neoplastic and neoplastic pathologies and plays a key role in staging [21, 23, 24]. In our study, of the 149 samples where the BMS had recorded a diagnostic impression, they were able to diagnose accurately with a sensitivity of 87.83%; this is similar to reported sensitivity levels of cytopathologists in other studies [21, 23–25]. This study demonstrated that an experienced BMS (as defined above) can reliably provide a diagnostic impression during ROSE for EBUS samples without loss of tissue for further immunohistochemistry staining. We could only find one other publication where the role of the BMS in ROSE in EBUS has been evaluated for diagnostic accuracy, however this was a retrospective audit where the preliminary diagnosis made by the BMS was submitted to the cytopathologist prior to their analysis which may introduce reporting bias [22].

Our study has highlighted that if a BMS diagnostic impression from ROSE was utilised for preliminary impression and fast-tracked to the cytopathologist, it could potentially reduce the burden on the lung cancer pathway in our institution. The current target is 28 days from referral as per the National Lung Cancer Pathway. Given it took an average of 9.59 days to reach a “malignant” diagnosis post EBUS procedure, this often equates to re-discussion in lung MDT up to 2 weeks after procedure to review these results. If the BMS was supported to provide an impression “real-time,” rather than just “sample adequacy” as per current guidelines, it would likely have a positive impact in many institutions, depending on local reporting times, days of the week that EBUS lists are performed, and presence of cytopathologists on weekends. With advances in telepathology and remote reporting there is potential to develop live streaming of slides with rapid stains from BMS to cytopathologists, who may be working remotely. Even though further molecular analysis may be required for malignant diagnosis, there are a substantial proportion of non-malignant diagnosis made (41/76 at site 1 and 31 out of 70 at site 2) and the potential to triage these patients to appropriate clinics, e.g., tuberculosis (TB) post-EBUS, is arguably not maximised given current reporting guidelines for BMS [19].

This data supports calls by the Cytopathology Conjoint Board – a joint committee of the Institue of Biomedical Science (IBMS) and the Royal College of Pathologists (RCPath) - alongside the British Association for Cytopathology (BAC), to permit trained BMS to provide a diagnostic impression instantly via ROSE. The proposition to utilising a BMS to aid diagnosis and guide further cytological molecular analysis demonstrates potential benefit to the health economy. Assuming that most centres would be using the service one whole day per week, the estimated cost of ROSE would be approximately £11,469.80 per annum (senior band 7/8a BMS salary up to £57,349/annum, working 0.2 whole time equivalent). It is worth noting that this may be significantly cheaper than previous cost analysis which may have assumed a ROSE service led by a cytopathologist, with consultant time being comparatively more costly.

Supporting BMS staff to become independent practitioners was highlighted in the RCPath 2017 survey, which demonstrated only 3 per cent of histopathology departments felt they had enough staff to meet clinical demand [18, 26]. Cancer Research UK estimates that with an increasing ageing population, the number of all cancer cases is predicted to rise by 40% by 2035, further increasing the demand on pathology services and this may prove to be critical with the current recruitment crises within the speciality, combined with an ageing workforce and potential retirement crisis [18]. The use of ROSE as a diagnostic tool has the potential to decrease the burden on processing time and materials for molecular analysis by streamlining the analysis process. Looking to the future, more trained BMS could assist in the development of digital techniques and pathways, for example, the use of telepathology for rapid off-site assessment, which may also facilitate training, and development of potential AI processes, where preparation and uploading of slides is key. Currently progression is limited despite accumulating data highlighting that with appropriate training and experience for BMS they can provide an impression more than just adequacy assessment during ROSE. The importance of training and experience is highlighted in the discrepancy in results between the two sites, where Site 1 (more EBUS procedures performed) demonstrated a sensitivity of 94.7% and specificity of 87,8%. However, site 2 had a sensitivity of BMS reporting of 79.5% and specificity of 77.4%. This demonstrates that results are not yet uniformly good enough across institutions to warrant sole BMS reporting of rapid stains at ROSE. However, training and experience is variable and not currently standardised, but with greater support they could be developed. Given the changing landscape with potential lung cancer screening and demand this may place on EBUS and pathology services [27], particularly for non-malignant cases, this may be an area worthy of further larger scale studies.

Meta-analysis in the “Guidelines for acquisition and preparation of EBUS samples” published by Van der Heijden et al. demonstrated that ROSE decreases the number of additional procedures needed to establish the diagnosis [5–8, 13]. Despite this data a recent RCPath 2020 cytopathology survey demonstrated that only 38% of laboratories within the UK offer ROSE as a service [26]. This data demonstrates that the current number of BMS reporting adequacy for ROSE with significant experience is low, training and standardisation to report adequacy of ROSE is not yet securely defined in BMS practice [19]. Schact et al found an inter-observer agreement between BMS and cytopathologist of >95% and further supports re-defining the role of the BMS in ROSE for EBUS-TBNA [22].

This study demonstrates the need within the pathology community to create a joint adequacy and diagnostic pathway for BMSs. A movement of pathology teams for EBUS-TBNA ROSE would encourage a collegiate cancer pathway with personal interaction with the laboratory team and the clinician. Within Europe there appears to be wide variability in practice as to who reports ROSE and there is insufficient evidence to conclude who should report, i.e., pathologists; cytopathologists; cytotechnicians, pulmonologists or trained nurses? [13]. There is however emerging evidence that trained respiratory physicians can perform ROSE, confirm adequacy and distinguish between malignancy and other pathologies and make a preliminary diagnosis [28, 29]. Although physicians or BMS could never replace the expert training and experience and the final impression of a consultant pathologist, our findings suggest there is room for expanding roles to support more efficient pathways and ease the burden of our pathology colleagues.

### Limitations

The study occurred where ROSE is routinely used, it was present for 75% EBUS-TBNA and therefore an element of bias cannot be excluded. The statistical power of this study is low due to the number of participants. Prospective reporting by the BMS was only performed in 149 cases and we would suggest a larger trial and national review into utilising BMSs to diagnostically interpret ROSE EBUS-TBNA to further validate this data. There is currently no agreed national standard for defining sample adequacy and although we have agreed local standards which conform to the limited literature in this area, this may vary nationally.

## Conclusion

This study represents the potential for utilising a BMSs diagnostics impression during EBUSTBNA with ROSE to enhance future fast track or “off-site” collaborative reporting pathways between BMS and Cytopathologists. The data provides an opportunity to increase training and experience for BMS to improve streamlining patients to appropriate services. It is unclear if previous cost analysis literature on the value of ROSE involved using cytopathologists or biomedical scientists on site, so further cost analysis to develop such pathways is key with further evaluation of how this may support the demands and recruitment crises within pathology services. Further studies into the accuracy of experienced BMSs reporting small cell lung cancer versus non-small cell lung cancer would be valuable and has the potential to further improve timely access to treatment.

## Summary Table

### What Is Known About This Topic

• Biomedical scientists (BMS) can report sample adequacy during EBUS TBNA using rapid on-site evaluation (ROSE).
• Despite being able to report exfoliative samples such as pleural fluid cytology and bronchial washings, they are usually not permitted to offer a preliminary diagnostic impression of EBUS TBNA samples.

### What This Work Adds

• Experienced biomedical scientists can provide a reliable diagnostic impression during ROSE for EBUS samples, with sensitivity and specificity comparable to cytopathologist reporting.
• This work represents an advance in biomedical science because it provides evidence a BMS can safely and accurately provide a real time cytopathological impression from EBUS TBNA sampling, which could positively impact patient pathways.

## Data Availability

The raw data supporting the conclusions of this article will be made available by the authors, without undue reservation.
